# Man Described

**Published:** 1869-05

**Authors:** 


### MAN DESCRIBED.

Chemically speaking, he is a pailful of water and a pound or two of ashes mixed up. He is defined in the Christian Examiner thus: "Regarded from a scientific point of view, the being of a man is a metamorphosis of the organic material of the world into forms of human tissues, and of the animating force of the world into the conditions of human consciousness." What is a dog? asked we of our little Robbie one day. In the plenitude of conscious mental might and right, he promptly replied, rising in his seat in his enthusiasm, "A dog is an animal with four legs and one tail."

